# In vitro host range, multiplication and virion forms of recombinant viruses obtained from co-infection in vitro with a vaccinia-vectored influenza vaccine and a naturally occurring cowpox virus isolate

**DOI:** 10.1186/1743-422X-6-55

**Published:** 2009-05-12

**Authors:** Malachy Ifeanyi Okeke, Øivind Nilssen, Ugo Moens, Morten Tryland, Terje Traavik

**Affiliations:** 1 Department of Microbiology and Virology, Faculty of Medicine, University of Tromsø, N-9037 Tromsø, Norway; 2 GenØk-Centre for Biosafety, Tromsø Science Park, N-9294 Tromsø, Norway; 3 Department of Medical Genetics, Institute of Clinical Medicine, University of Tromsø, N-9037 Tromsø, Norway; 4 University Hospital of North-Norway, N-9038 Tromsø, Norway; 5 Department of Food Safety and Infection Biology, The Norwegian School of Veterinary Science, N-9010 Tromsø, Norway; 6 Institute of Pharmacy, Faculty of Medicine, University of Tromsø, N-9037 Tromsø, Norway

## Abstract

**Background:**

Poxvirus-vectored vaccines against infectious diseases and cancer are currently under development. We hypothesized that the extensive use of poxvirus-vectored vaccine in future might result in co-infection and recombination between the vaccine virus and naturally occurring poxviruses, resulting in hybrid viruses with unpredictable characteristics. Previously, we confirmed that co-infecting in vitro a Modified vaccinia virus Ankara (MVA) strain engineered to express influenza virus haemagglutinin (*HA*) and nucleoprotein (*NP*) genes with a naturally occurring cowpox virus (CPXV-NOH1) resulted in recombinant progeny viruses (H Hansen, MI Okeke, Ø Nilssen, T Traavik, Vaccine 23: 499–506, 2004). In this study we analyzed the biological properties of parental and progeny hybrid viruses.

**Results:**

Five CPXV/MVA progeny viruses were isolated based on plaque phenotype and the expression of influenza virus HA protein. Progeny hybrid viruses displayed in vitro cell line tropism of CPXV-NOH1, but not that of MVA. The *HA *transgene or its expression was lost on serial passage of transgenic viruses and the speed at which HA expression was lost varied with cell lines. The HA transgene in the progeny viruses or its expression was stable in African Green Monkey derived Vero cells but became unstable in rat derived IEC-6 cells. Hybrid viruses lacking the *HA *transgene have higher levels of virus multiplication in mammalian cell lines and produced more enveloped virions than the transgene positive progenitor virus strain. Analysis of the subcellular localization of the transgenic HA protein showed that neither virus strain nor cell line have effect on the subcellular targets of the HA protein. The influenza virus HA protein was targeted to enveloped virions, plasma membrane, Golgi apparatus and cytoplasmic vesicles.

**Conclusion:**

Our results suggest that homologous recombination between poxvirus-vectored vaccine and naturally circulating poxviruses, genetic instability of the transgene, accumulation of non-transgene expressing vectors or hybrid virus progenies, as well as cell line/type specific selection against the transgene are potential complications that may result if poxvirus vectored vaccines are extensively used in animals and man.

## Background

The family *Poxviridae *consists of large double stranded DNA viruses that replicate in the cytoplasm of infected cells [1,2]. Within this family, vaccinia and cowpox viruses are members of the genus *Orthopoxvirus*. Poxviruses are increasingly being used as vectors for efficient gene expression in vitro and in vivo [2-4]. The future use of poxvirus vectors for delivery of prophylactic and therapeutic vaccines has raised potential biosafety concerns. Putative risks associated with the use of genetically modified poxviruses as vaccines include virulence of the vector, stability of inserted transgene, potential transmission to non-target species and recombination between the vaccine vector and a naturally circulating poxvirus [5,6]. The risks of virulence and spread to non-target species have been addressed in part by the use of attenuated strains like modified vaccinia virus Ankara (MVA). MVA multiplication seems to be restricted in most mammalian cells. So far it has only been shown to carry out full productive infections in BHK-21 and IEC-6 cells respectively [7,8]. MVA is considered apathogenic even when administered in high doses to immune deficient animals [9-11]. Several MVA vectored vaccines against infectious diseases and cancers are in various phases of field and clinical trials [12-16].

MVA can be genetically modified by recombination with a naturally occurring wild type orthopoxvirus (OPV) during mixed infection. Alternatively, the transgene in the MVA vector can be recombined into a replication competent poxvirus during co-infection. To assess the risk of recombination, it is essential that the MVA vector and a naturally circulating poxvirus co-infect the same cell or host. The potential widespread use of MVA vectored vaccines (especially in wild-life and free ranging domestic animals), and therapeutic vaccination with MVA against emerging OPV epidemics are likely scenarios for mixed infection between vaccine strains of OPVs and naturally circulating relatives. Post exposure application of MVA to treat pre-existing OPV infection is a likely scenario for co-infection. Indeed post exposure application of MVA has been shown to protect against lethal OPV infection [17]. Poxviruses undergo a high frequency of homologous recombination in the cytoplasm of infected cells [18-22]. Poxvirus recombination, which is inextricably linked with DNA replication requires 12 bp end sequence homology between the recombinogenetic templates [23,24]. Thus, even the highly attenuated MVA can undergo homologous recombination in non-permissive cells or hosts since DNA replication is unimpaired. Although homologous recombination is the method of choice for generating transgenic MVA vectored vaccines [13], studies on recombination between transgenic MVA vectors and wild type poxviruses are miniscule. Analysis of co-infection and recombination between MVA vectored vaccines and wild type OPVs is a safer model for evaluating the potential consequences of recombination between poxvirus vectored vaccines and naturally circulating OPVs than using multiplication competent poxvirus vectors. In addition, the characterization of hybrid progenies arising from recombination between transgenic MVA and wild type OPVs will provide valuable information on poxvirus host range, morphogenesis, cytopathogenicity (CPE), replication fitness, transgene stability and transgenic protein localization.

Previously, we have isolated and genetically mapped recombinant viruses obtained from co-infection of cells with a transgenic MVA strain (MVA-HANP) engineered to express the influenza virus haemagglutinin (*HA*) and nucleoprotein (*NP*) genes and a naturally circulating cowpox virus (CPXV-NOH1) [6]. In the present study we analyzed the biological properties of parental and progeny hybrid viruses. We show that the transgene or its expression was lost following serial passage of some of the hybrid viruses in mammalian cells, and that the resulting transgene negative virus strains have an enhanced virus multiplication in several mammalian cell lines. In addition, the stability of the transgene or the loss of its expression varies with cell lines used for virus multiplication.

## Results

### Cell line permissivity, cytopathogenicity and plaque phenotypes

**Figure 1:**
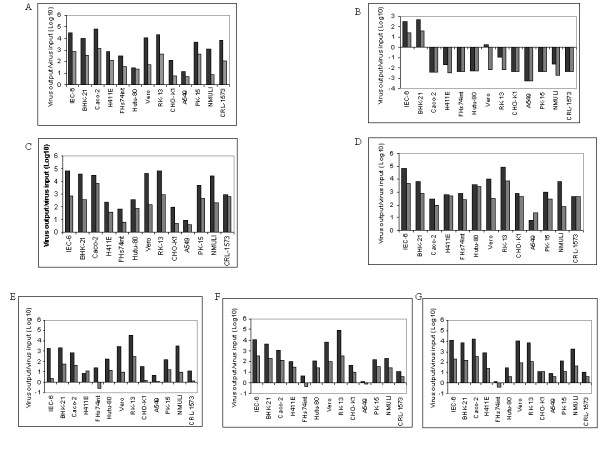
**Multiplication of parental and progeny viruses in mammalian cell lines**. Virus multiplication (fold increase in virus titre) was determined by dividing the virus titre at 72 hpi by virus titre after adsorption. Black bars (virus multiplication in the cell); grey bars (virus in the culture medium). The values are mean of two independent experiments titrated in duplicate. CPXV-NOH1 (A), MVA-HANP (B), Rec 1 (C), Rec 2 (D), Rec 3 (E), Rec 3a (F), Rec 3b (G).

To investigate the in vitro host range, cytopathogenicity and plaque phenotypes of parental and progeny viruses, thirteen mammalian cell lines from various species and tissues were infected with viruses under study. The parental CPXV-NOH1 has a broad host range and multiplied in all the cell lines (Figure 1A). Caco-2, RK-13, IEC-6 and Vero cells supported high virus multiplication while virus production was lower in A549, CHO-K1 and Hutu-80 cells (Figure 1A). MVA-HANP multiplied only in IEC-6 and BHK-21 cells (Figure 1B). Detailed analysis of MVA multiplication and morphogenesis was described in a previous report [7]. The in vitro host range of Rec 1 is similar to CPXV-NOH1 except that higher levels of virus multiplication were obtained in many cell lines (Figure 1C). Rec 2 underwent productive infection in all the cell lines. However, unlike CPXV-NOH1 and Rec 1, its multiplication was characterized by high production of extracellular virions (Figure 1D). In fact, in A549 cells more virions were released to the medium than intracellular virus particles (Figure 1D). Compared to CPXV-NOHI, Rec 1 and Rec 2, Rec 3 had reduced virus multiplication in all the cell lines (Figure 1E). The transgene negative derivatives of Rec 3 (Rec 3a and Rec 3b) had in vitro host ranges comparable to Rec 3, except that virus production was more efficient (Figures 1F, G). Unlike CPXV-NOH1, Rec 1 and Rec 2, no extracellular virions were detected in FHs74int cells infected with Rec 3 and its transgene negative derivatives 3a and 3b (Figure 1).

**Figure 2:**
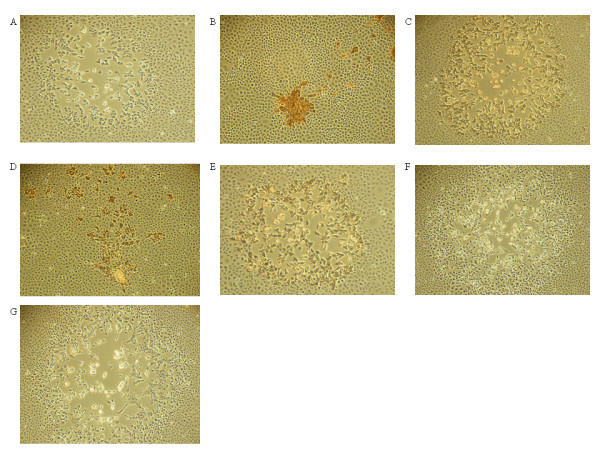
**Plaque phenotypes of parental virus strains and hybrid virus progenies in IEC-6 cells**. Confluent IEC-6 cells were infected with the respective viruses and the HA expression was monitored at 36 hpi by immunoperoxidase staining of fixed cells. The panels show representative fields at approximately × 200 magnification. CPXV-NOHI (A), MVA-HANP (B), Rec 1(C), Rec 2 (D), Rec 3 (E), Rec 3a (F), Rec 3b (G).

CPXV-NOHI produced low, moderate and high CPE in one, seven and five cell lines respectively (Table 1). MVA-HANP gave low or no CPE in all the cell lines except BHK-21 and IEC-6 cells where it produced high and moderate CPE, respectively. Rec 1 had moderate to very high CPE in all the cell lines tested except Caco-2. Compared to parental CPXV-NOH1, Rec 1 showed enhanced CPE in seven cell lines (Table 1). Similarly, Rec 2 resulted in higher CPE in most cell lines compared to parental CPXV-NOHI. Conversely, Rec 3 resulted in lower CPE compared to CPXV-NOHI in most cell lines except RK-13 and CHO-KI cells. Interestingly, transgene negative derivatives of Rec 3 (Rec 3a and Rec 3b) produced higher CPE in many cell lines compared to the transgene positive Rec 3 (Table 1). In particular, Rec 3b is the most cytopathogenic of virus strains investigated in this study. The plaque phenotypes of parental and progeny viruses were examined in thirteen mammalian cell lines. Previously, we have reported the plaque phenotypes of these viruses in Vero cells [6]. However, MVA does not form distinct plaques in Vero cells and thus comparison of plaque phenotypes of hybrid viruses was made only with the parental CPXV-NOH1 [6]. Therefore, we re-examined plaque phenotypes of parental and hybrid viruses in rat IEC-6, a cell line in which MVA forms very clear plaques [7]. CPXV-NOH1 produced large lytic plaques in IEC-6 cells (Figure 2A) and the other twelve cell lines (data not shown). In permissive IEC-6 cells, MVA-HANP plaques were small, non-lytic with characteristic comet (satellite) formation (Figure 2B). The plaque phenotype of Rec 1 in IEC-6 cells (Figure 2C) and other cell lines (data not shown) was similar to CPXV-NOHI except that plaques were larger in size. Rec 2 produced small plaques and comets in IEC-6 cells (Figure 2D). Comet formation was enhanced in Rec 2 compared to MVA-HANP although the size of the primary plaque is larger in the latter. Rec 3 produced large semi-lytic plaques with some undetached cells in the center of the plaque (Figure 2E). Rec 3a plaques were very large and lytic (Figure 2F). Rec 3b produced the largest plaque size in IEC-6 cells (Fig. 2G) and other cell lines and its plaques were characterized by high level of cell detachment and syncytia formation. Taken in tandem, the progeny viruses displayed parental and non-parental characteristics with respect to in vitro host range, CPE and plaque phenotypes.

**Table 1:** Cytopathic effects (CPE) produced by parental and progeny virus strains in mammalian cell lines.

Cytopathic Effects (CPE)^a^								
Cell line	Species/tissue	CPXV-NOH1	MVA-HANP	Rec1	Rec 2	Rec 3	Rec 3a	Rec 3b

IEC-6	Rat/small intestine; normal	+++	++	+++	++++	++	++++	++++
BHK-21	Hamster syrian/kidney; normal	++	+++	++++	++++	++	++++	++++
Caco-2	Human/colon; colorectal adenocarcinoma	+++	-	+	++	+++	+++	++++
H411E	Rat/liver; hepatoma	++	+	++	+++	+	+++	+++
FHs74int	Human/small intestine; normal	+++	-	++	+	++	+++	++++
Hutu-80	Human/duodenum; adenocarcinoma	++	-	++++	++++	++	++++	++++
Vero	African Green Monkey/kidney; normal	+++	+	++++	++	+	++++	++++
RK-13	Rabbit/kidney; normal	++	+	++++	+++	++++	+++	++++
CHO-K1	Hamster Chinese/ovary	++	-	+++	++++	+++	+++	+++
A549	Human/lung; carcinoma	+	-	++	++	+	++	++
PK15	Pig/kidney; normal	++	+	++	++	+	++	+++
NMULI	Mouse/liver; normal	++	-	+++	+++	+	++++	++++
HEK-293 (CRL-1573)	Human/kidney; transformed with adenovirus 5 DNA	+++	-	+++	++	+	++	++

^a ^Virus infection was at a m.o.i of 0.05 pfu per cell. CPE was categorized on the following criteria: no difference from uninfected cells (-); low, < 25% CPE (+); moderate, 25 to 50%CPE (++); high, > 50 to 75% CPE (+++); very high, > 75 to 100% CPE or high level of cell detachment (++++) [25].

### Virus multiplication at low and high multiplicity of infection (m.o.i)

**Figure 3:**
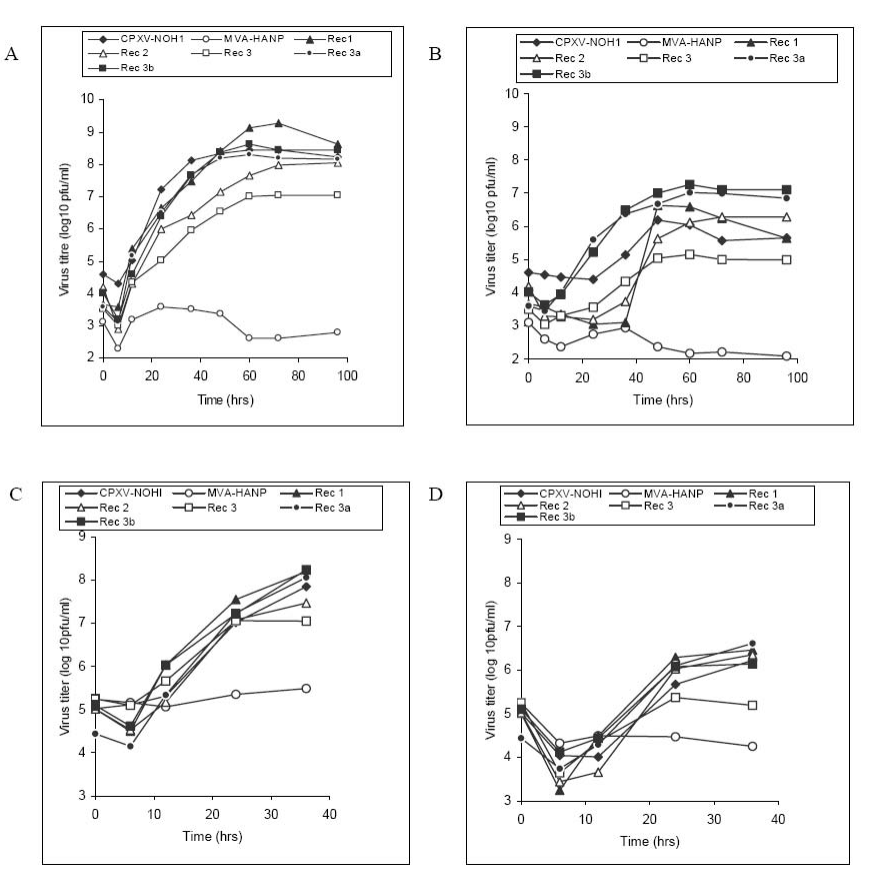
**Time course of virus production in Vero cells at low and high multiplicity of infection**. Confluent Vero cells were infected with the respective virus strains at low m.o.i (0.01 pfu per cell) and high m.o.i (5.0 pfu per cell). Virus production in the cell (A) and virus released to the supernatant (B) at low m.o.i. Virus production in the cell (C) and virus released to the culture medium (D) at high m.o.i. Values are means of two independent experiments titrated in duplicates.

Multistep multiplication at low m.o.i and single step multiplication at high m.o.i are standard methods for quantifying infectious virus production [25]. The low m.o.i analysis was carried out at a m.o.i of 0.01 pfu per cell. The low m.o.i kinetics of virions produced in the cell and liberated into the medium is summarized in Figures 3A and 3B. At low m.o.i, CPXV-NOHI virion production in the cell increased exponentially after a short lag period, reaching 8.35 logs at 60 hpi (Figure 3A). Release of virions into the medium was inefficient and was characterized by 24 hours lag period and low virus yield (Figure 3B). MVA-HANP multiplied poorly in Vero cells (Figures 3A, B). Intracellular virus production in Rec 1 was very high as evidenced by very high titre (9.12 logs) and yield (5.4 logs). Although the release of Rec 1 virions from Vero cells infected at low moi was delayed (36 hpi) compared to other strains that already released virons (20 hpi or less), it was a short and spontaneous single burst (Figure 3B). This suggested that Rec 1 is a very lytic virus. Rec 2 form small plaques but virion production appears unhindered. Intracellular virus multiplication was gradually reaching a titer of 8 logs at late times post infection (Figure 3A). Expectedly, 35% of Rec 2 total infectivity was liberated into the medium (Figure 3B). This is not surprising since Rec 2 is very efficient in producing comets. Multiplication kinetics of Rec 3 showed that virion production or its spread was less efficient than other strains infected at lower m.o.i. Intracellular and extracellular virions produced by Rec 3 were approximately 1 log or more lower than that of other strains (Figures 3A, B). Interestingly, the HA negative viruses (Rec 3a and Rec 3b) derived from Rec 3 by the spontaneous deletion of the *HA *following serial passage in Vero cells [6] showed improved levels of virus multiplication than their ancestor. Indeed at various time points post infection, Rec 3a and Rec 3b virus titre (in the cell and medium) were at least one log higher than Rec 3 (Figures 3A, B). At this juncture, we do not know the reason for the increase in virus production in the *HA *negative Rec 3a and Rec 3b.

Virus production and spread is influenced by the m.o.i. Thus low m.o.i multi step conditions may generate different multiplication profile from synchronized single step conditions at high m.o.i [25]. Thus we generated multiplication profiles of test virus strains following synchronized infection at a m.o.i of 5 pfu per cell. The results are summarized in Figures 3C and 3D. CPXV-NOHI has similar multiplication kinetics with all progeny viruses from adsorption to 24 hpi, although Rec 3 has the lowest intracellular and extracellular virus titres (Figures 3C, D). MVA-HANP performs limited virus multiplication in Vero cells. Consistent with what was obtained under multi-step conditions, transgene negative progenies of Rec 3 (Rec 3a and Rec 3b) have higher levels of virus multiplication compared to Rec 3 (Figures 3C, D). Thus, compared to the *HA *positive progenitor strain (Rec 3), the *HA *negative derivatives (Rec 3a and Rec 3b) have enhanced virus multiplication at both low and high m.o.i.

### Stability of the transgene in mammalian cells

**Figure 4:**
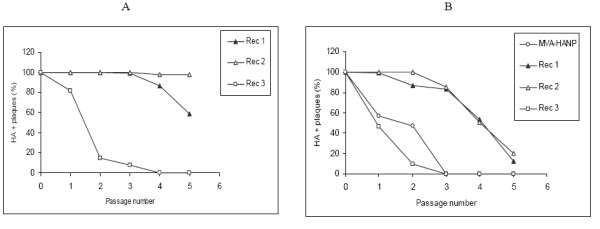
**Stability of the HA transgene in mammalian cell lines**. The stability of the influenza virus transgene inserted into MVA and hybrid progenies was assayed indirectly by monitoring the HA phenotype. Serial passage of transgenic virus strains in Vero (A) and IEC-6 (B) cells.

Since virus tropism is dependent on the host or cell type, we hypothesized that the stability of the influenza virus *HA *insert in the transgenic viruses may vary in different cell types or lines. To our knowledge, the stability of the transgene in MVA vectored vaccines in different cell types or hosts has not been reported. To address this hypothesis, transgene positive viruses were passaged in Vero and IEC-6 cells for five times at a m.o.i of 0.01 pfu per cell. Consistent with our previous report, the HA phenotype of Rec 3 was unstable in Vero cells [6]. By the 4th passage, HA + plaques were undetected in Vero cells (Figure 4A). The HA phenotype of Rec 1 in Vero cells was stable up to passage 3. However, by passage 5, only 58% of Rec 1 plaques were HA + (Figure 4A). The HA + phenotype of Rec 2 was very stable in Vero cells across several passages (Figure 4A). These results suggested varying degrees of stability of the transgene in the progeny transgenic viruses. The stability of the HA + phenotype of MVA-HANP in Vero cells was not included because of low virus titres. The HA + phenotype of all transgenic viruses including MVA-HANP was unstable in IEC-6 cells (Figure 4B). The highest level of instability was observed with MVA-HANP and Rec 3. In both viruses, the HA + plaques were undetectable at passage 3 (Figure 4B). Beyond passage 3, the HA + phenotype of Rec 1 and Rec 2 were unstable to the extent that approximately 20% of the plaques were HA + at passage 5 (Figure 4B). Also, the reduction in the number of HA expressing viruses (MVA-HANP, Rec 3) was accompanied by a dramatic increase in the number of none HA expressing viruses (data not shown). There was no significant variation in the titre of each transgenic virus in Vero or IEC-6 cells at each serial passage (data not shown). Thus, these results suggest that the cell line or cell type used for virus multiplication might influence the stability of the transgene inserted into poxvirus vectors or determine the speed at which the expression of the transgene is lost. In addition, it shows the selection and accumulation of virus mutants that have lost the transgene or its expression.

### Shape and size of virions

**Figure 5:**
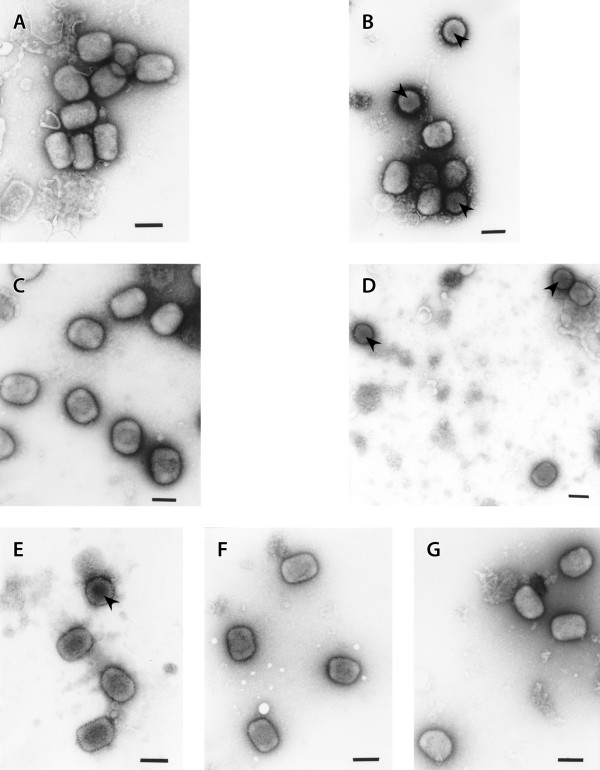
**Negatively stained purified virions of parental virus strains and hybrid progenies**. CPXV-NOHI (A), MVA-HANP (B), Rec 1 (C), Rec 2 (D), Rec 3 (E), Rec 3a (F), Rec 3b (G). Arrows (round virions). Bars, 200 nm (A-G).

The shape and size of negatively stained purified virions were determined in order to ascertain whether there are differences in the virion 2D architecture of the virus strains under study. The results are shown in Figure 5 and Table 2. The virions of CPXV-NOHI were brick shaped measuring 293 ± 27 nm × 229 ± 23 nm in size (Figure 5A, Table 2). Virions of CPXV-NOH1 were slightly smaller than what has been reported for strains of vaccinia virus [26-28]. Conversely, half of the virions of MVA-HANP were brick shaped (314 ± 23 nm × 256 ± 18 nm) while the other half were round shaped with dimensions measuring 255 ± 28 nm × 243 ± 29 nm (Figure 5B, Table 2). Virions obtained from Rec 1, Rec 3, Rec 3a and Rec 3b resemble that of CPXV-NOH1 in being mostly brick shaped. Unlike CPXV-NOH1, a small percentage of virions obtained from the aforementioned progeny viruses have round shape (Figure 5, Table 2). Apparently the virions of Rec 2 appear to be a mixture of what was obtained from the parental strains. Two thirds of Rec 2 virions were brick shaped and the remaining one third were round in shape (Figure 5D, Table 2). The results indicated that the brick shape is the major virion shape in all virus strains except MVA-HANP.

**Table 2:** Shape and size of negatively stained purified virions.

Shape and dimensions of purified virions^a^						
Virus strain	Brick			Round		
	N (%)	Length (nm)	Width (nm)	N (%)	Length (nm)	Width (nm)

CPXV-NOH1	50 (100)	293 ± 27	229 ± 23	0 (0)	-	-
MVA-HANP	25 (50)	314 ± 23	256 ± 18	25 (50)	255 ± 28	243 ± 29
Rec 1	43 (86)	303 ± 17	244 ± 15	7 (14)	272 ± 35	264 ± 35
Rec 2	32 (64)	287 ± 15	237 ± 16	18 (36)	253 ± 19	238 ± 18
Rec 3	42 (84)	300 ± 18	235 ± 12	8 (16)	251 ± 15	238 ± 18
Rec 3a	43 (86)	292 ± 15	229 ± 14	7 (14)	257 ± 11	243 ± 8
Rec 3b	47 (94)	302 ± 13	240 ± 13	3 (6)	257 ± 6	243 ± 6

^a ^50 virions from each virus strain were randomly selected and their shapes and sizes determined.

### Virus morphogenesis

**Figure 6:**
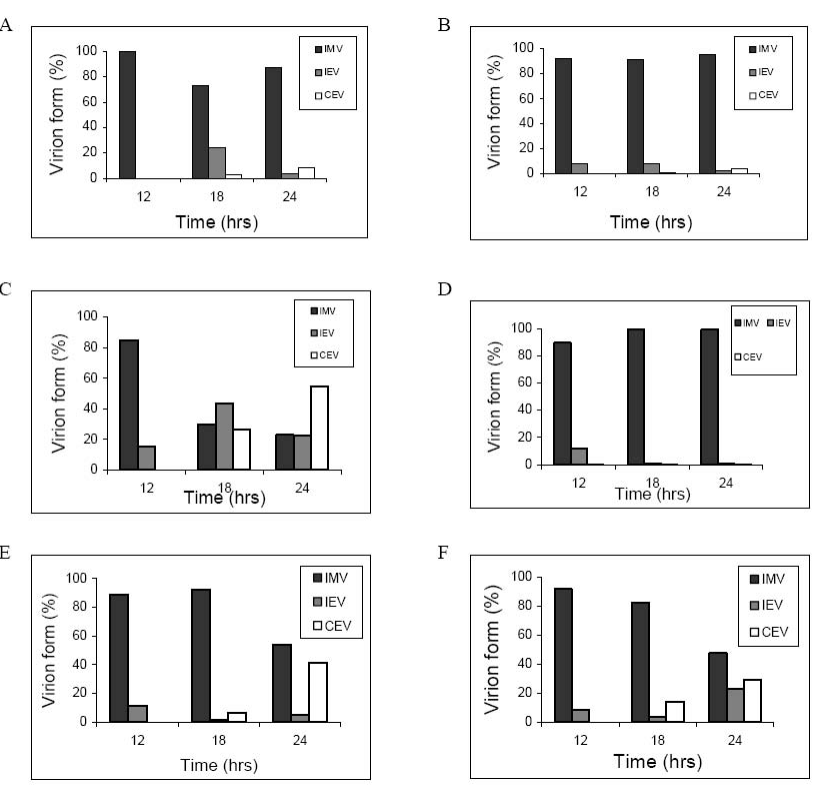
**Relative amount of mature virus forms produced by respective virus strains in Vero cells**. Virion forms produced at different times post infection were quantified by electron microscopy as described in methods. IMV, IEV and CEV were counted in 50 randomly chosen sections of infected cells. The values represent percentage of each virus form relative to the total number of virus forms counted. IMV, intracellular mature virus; CEV, cell associated enveloped virus; EEV, extracellular enveloped virus. CPXV-NOHI (A), Rec 1 (B), Rec 2 (C), Rec 3 (D), Rec 3a (E), Rec 3b (F).

We carried out detailed analysis of the morphogenesis of the virus strains under study by electron microscopy. Relative and absolute numbers of various mature and immature viral forms were determined at different times post infection. The kinetics of mature virus production in Vero cells is depicted in Figure 6. MVA-HANP results were not included because a very low level of mature virus forms were produced in Vero cells [7]. Assembly of CPXV-NOHI and hybrid progenies was similar to what have been reported for strains of vaccinia virus [7,29,30]. Differences existed in the abundance of mature virus forms produced by CPXV-NOHI and progeny viruses. The intracellular mature virus (IMV) is the major mature virus type produced in CPXV-NOH1 infected cells accounting for 87% of virion forms (IMV, IEV, CEV) at 24 hpi (Figure 6A). Intracellular enveloped virus (IEV) and cell associated enveloped virus (CEV) represented 4% and 9% respectively of virion forms produced in CPXV-NOH1 infected cells (Figure 6A). Similarly, 95% of virions produced by Rec 1 at 24 hpi were IMVs (Figure 6B). However in Rec 2, a higher proportion of IMVs were converted to enveloped forms such that at 24 hpi, CEV is the predominant form representing 55% of virion forms produced (Figure 6C). Rec 3 appeared defective in the production of enveloped virions. At 18 and 24 hpi, less than 1% of Rec 3 virions were IEV or CEV (Figure 6D). Transgene negative derivatives of Rec 3 (Rec 3a and Rec 3b) were efficient in the production of enveloped virions. The enveloped forms (IEV and CEV) accounted for approximately 50% of virion types produced by Rec 3a and Rec 3b respectively at 24 hpi (Figures 6E, F).

### Localization of the transgenic protein

**Figure 7:**
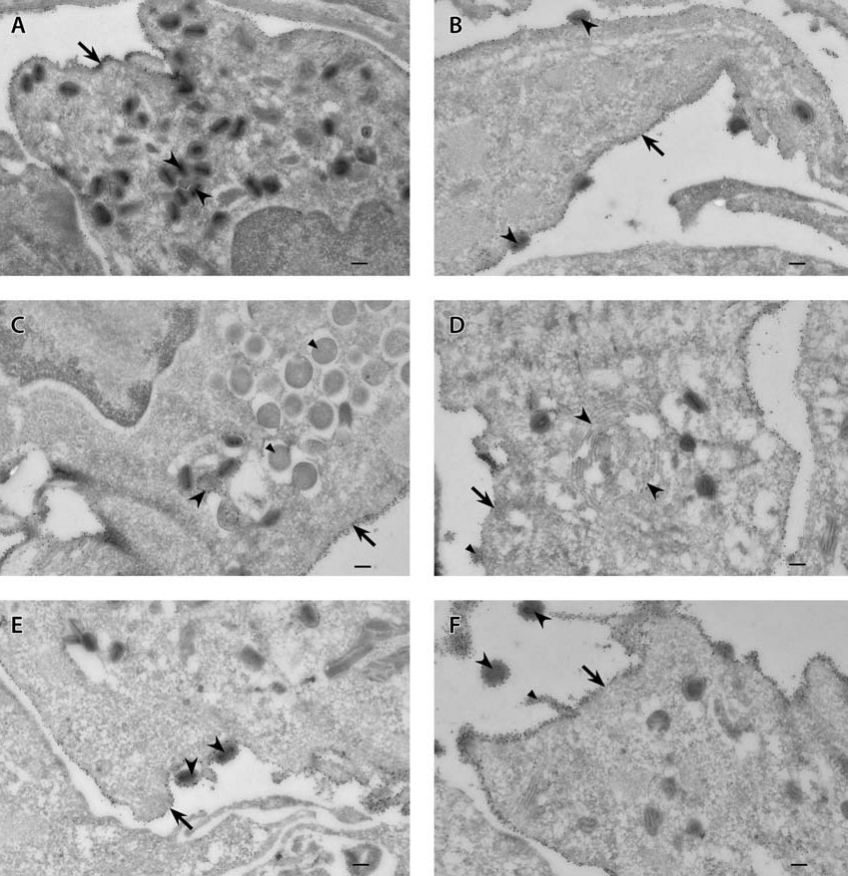
**Cellular and viral localization of the influenza virus haemagglutinin protein**. Vero cells were infected with MVA-HANP and HA + hybrid progenies and processed for immunoelectron microscopy as detailed in methods. Rec 1 infected Vero cells (A, B) and Rec 2 infected Vero cells (C-F). (A): arrow (plasma membrane), arrow heads (IMVs); (B): arrow (plasma membrane), arrow heads (CEVs); (C): arrow (plasma membrane), large arrow head (trans-Golgi membrane), small arrow heads (immature viruses); (D): arrow (plasma membrane), large arrow heads (Golgi apparatus), small arrow (cell associated vesicle); (E): arrow (plasma membrane), arrow heads (CEVs); (F): arrow (plasma membrane), large arrow heads (EEVs), small arrow head (plasma membrane projection). The same results were obtained with MVA-HANP and Rec 3 (data not shown). IMV, intracellular mature virus; CEV, cell associated enveloped virus; EEV, extracellular enveloped virus. Bars, 100 nm (A-F).

We used immunogold cryo electron microscopy to track the localization of influenza virus HA protein produced by transgenic poxviruses in infected cells. The cellular and viral location of the transgenic protein was the same for all transgenic viruses (MVA-HANP, Rec 1, Rec 2, Rec 3), and was also independent of cell line used for virus cultivation. The transgenic protein was absent in IMVs and immature viruses (Figures 7A, C). The influenza virus HA protein was concentrated on the plasma membrane (Figures 7A–F), Golgi apparatus (Figure 7D), trans-Golgi membrane (Figure 7C), CEVs (Figures 7B, E), and EEVs (Figures 7F). Gold particles were also present in cell-associated vesicles (Figure 7D), vacuoles, cytoplasmic vesicles and exocytic vesicles (data not shown). Overall, the influenza virus HA protein was targeted to enveloped virions and cellular compartments associated with the down stream exocytic pathway. Also the transgenic HA protein targets are independent of virus backbone expressing the transgene as well as the cell line used for virus multiplication.

## Discussion

In this work we have investigated biological properties of progeny hybrid viruses obtained from recombination in vitro with a transgenic MVA candidate vaccine and a naturally circulating cowpox virus. The hypothesis of this work is that the extensive use of poxvirus-vectored vaccines in future might result in natural in vivo co-infection and recombination between poxvirus-vectored vaccines and naturally circulating orthopoxviruses resulting in hybrid viruses with non-parental characteristics. MVA and a naturally circulating cowpox virus were used as parental strains to test this hypothesis in vitro. MVA is arguably the vector of choice for antigen delivery [31], and the wide spread use of MVA vectored vaccines (especially in wild life and domestic animals) in the future is highly likely. Cowpox virus is the ancestor of other OPVs, has broad host range, and contains the most complete repertoire of immunomodulators [32-34]. Unlike vaccinia virus where DNAemia or viremia seems to be an extremely rare event in vaccinees [35], DNAemia in patients with localized symptoms of cowpox virus infection seem not to be a rare event [36]. Indeed cowpox virus DNA was detected in whole blood of two independent patients at 4 weeks post infection [36]. Persistence of cowpox virus DNA in infected individuals increases the likelihood of recombination during co-infection with a poxvirus-vectored vaccine. Thus homologous recombination between MVA vectored vaccine and a naturally circulating cowpox virus (or other orthopoxviruses) can occur and such a recombination has the potential of generating novel hybrid viruses that will elucidate our understanding of the biology of recombinant poxviruses, as well as the putative scenarios that might arise following the release of genetically modified poxviruses in the wild.

CPXV-NOH1 underwent productive infection in all the thirteen mammalian cell lines used in this study. This is consistent with the broad host range of other cowpox virus strains [37,38]. The progeny viruses have cell line tropism similar to CPXV-NOHI, but not to MVA-HANP. Although few host range genes have been identified in cowpox virus [39], sequence analysis of OPV genomes indicated that host range genes cluster at the genome termini [32,40]. Our previous work suggested that all the progeny viruses derived their genome termini from CPXV-NOH1 [6]. Thus the hybrid viruses might have derived their host range genes from CPXV-NOH1. With the exception of Rec 3, progeny viruses have higher levels of CPE in most mammalian cell lines than the parental strains. A possible explanation is that progeny viruses have a more effective mechanism for the shutdown of host protein synthesis.

Progeny viruses displayed plaque phenotypes different from parental strains. The plaque phenotypes of the progenies were reproducible in all the cell lines (data not shown). Large lytic plaques are often associated with efficient cell to cell spread in cell cultures while small plaques may indicate inefficient cell to cell spread [41,42]. The genetic basis of the plaque phenotypes in the parental and progeny viruses is unknown. However, in vaccinia virus Western Reserve (VACV-WR), it has been demonstrated that five EEV proteins (gene products of *A33R*, *A34R*, *A56R*, *B5R*, *F13L*) and two IEV proteins (A36R, F12L) may be involved in determining plaque phenotypes [43-49]. Although functionally intact EEV and IEV membrane proteins are associated with large plaque phenotype, they are insufficient in determining plaque size per se [50]. It has also been reported that the production of actin tails is the major factor correlating with plaque size [51]. Rec 2 plaques were characterized by the formation of comets. Comet formation was present, albeit to a lower degree in the parental MVA-HANP. Thus it is plausible that the genes for comet formation in Rec 2 were derived from MVA-HANP. In vaccinia virus IHD, comets are due to point mutation in the *A34R *open reading frame (ORF) or a second site mutation in the *A33R *and *B5R *ORFs [52,53]. Rec 3b plaques displayed high degree of syncytium formation, a trait not observed in the parental strains and other progeny viruses. Mutation in the *A56R *is known to cause syncytia in vaccinia virus [54].

Three experimental observations made in this study have potential relevance for the release of genetically modified poxviruses into the ecosystem as well as recombination between transgenic poxviruses and naturally occurring relatives. Firstly, the transgene is deleted at high frequency in Rec 3 and MVA-HANP probably as a result of adaptation to the cell lines. Secondly, the viruses that have lost the transgene have higher virus multiplication compared to the transgene positive progenitor strain. Thirdly, there is variation in the stability of the transgene or its phenotype in different cell lines. The HA phenotype of Rec 2 was very stable in African Green Monkey derived Vero cells but became unstable in rat derived intestinal IEC-6 cells. The loss of the transgene/transgene phenotype as part of adaptation to new cells or hosts, the subsequent positive selection and accumulation of none transgene expressing virus mutants might compromise the efficacy of poxvirus vectored vaccines. The loss of the transgene will likely result in less effective vaccine since there will be less antigen to elicit robust immune responses. However since MVA undergoes abortive infection in most mammalian cell types, the accumulation of none transgene expressing viruses in the vaccinated hosts seem unlikely. The loss of the transgene or its phenotype and subsequent accumulation of none transgene expressing vector will be a likely problem for poxvirus vaccines based on replication competent vaccinia virus. The apparent variation in the stability of the *HA *transgene or the HA phenotype across different cell lines raises the possibility that the transgene inserted into poxviruses may have varying stability in different hosts. Although, spontaneous deletion [6] and truncation [55] of transgenes have been observed in some candidate MVA vectored vaccines, this is the first report showing that the same MVA or CPXV/MVA vectored vaccines was stable in one cell line and unstable in the other. Thus, our findings suggest that there is a host cell selection against the transgene. We have no explanation for the differential loss of the HA expression in different cell lines and among different viruses following serial passage. However, we speculate that the structure of the inserted *HA *transgene, the promoters driving the HA expression, the direction of the inserted *HA *transgene relative to the surrounding genes, the inherent stability/instability of the genetic locus at which the *HA *was inserted and the host cell responses to the HA protein are some of the factors that might affect the stability of the *HA *transgene or the loss of the HA expression following serial passage of the transgenic viruses. Genome wide mapping of these recombinant viruses will shed light on the genetic basis for the biological observations made in this study.

The purified virions of CPXV-NOH1 are brick shaped with highly corrugated surface similar to the structure of VACV-WR [28]. The virions of MVA-HANP are pleomorphic with half of the virions being brick shaped and the other half round. The observation is in concert with a previous report [27]. A 3D reconstruction of VACV-WR virions claimed that all IMVs are brick shaped and the observation of varied shapes in earlier studies is due to the limitation of 2D imaging [26]. The round form observed in this study for MVA-HANP and the hybrid viruses (Figures 5B, D, E) is very close to a circle such that the difference between the length and width is 15 nm or less (Table 2). However, 2D measurements as done in this study can be affected by the angle of tilt as well as the plane at which the virions lie on the grids. Thus, our finding of spherical or round forms of virions especially in MVA need to be confirmed by 3D reconstruction of MVA and other OPV strains.

IMV is the major viral form produced in Vero cells infected with CPXV-NOH1. The dominance of IMVs over the enveloped forms may not be unconnected with the fact that CPXV-NOH1 produces V^+ ^A-type inclusion (ATI) in infected cells (data not shown). It has been suggested that IMVs marked for sequestration into ATI may not differentiate into IEVs, CEVs or EEVs [56]. Like CPXV-NOH1, IMVs constitute over 90% of virions produced in Vero cells infected with Rec 1 at various times post infection. However in Rec 2, the percentage of enveloped forms (IEV and CEV) is higher than that of IMV. Probably, the trans-Golgi network (TGN) wrapping and transport of IEV on microtubules is very efficient in Rec 2. Rec 3 on the other hand produces very low number of IEV or CEV and may be defective in the wrapping of IMV by the TGN. In Rec 3a and Rec 3b, the proportions of IMV and enveloped virions at 24 hpi were almost equal. Kinetics of virion formation in Rec 3a indicated high percentage of CEVs even though that of IEVs is low. It seems that some of the CEVs observed in Rec 3a infected cells were produced by plasma membrane budding of IMV rather than fusion of IEV outer envelope with the plasma membrane. Budding of IMVs through the plasma membrane has been shown to be an alternative mechanism for the production of CEVs [57].

The incorporation of influenza virus HA protein into the CEV and EEV of transgenic viruses has potential biosafety and immunological implications. Although foreign protein on the surface of CEVs and EEVs may enhance the humoral immune response of the host [58], they may also alter the host range or cell tropism of the transgenic MVA. Although MVA is host restricted and may not form sufficient CEVs/EEVs in human cells, the transgene on the MVA vector can be inserted by homologous recombination into another OPV with broad host range during mixed infection. Indeed we have shown in this work that the CEV and EEV of hybrid viruses incorporated the transgenic protein on their surface. We assume that the localization of the transgenic protein on CEV/EEV but not IMV or immature viruses is because the former derived its envelope from TGN or the plasma membrane [57,59]. Both the TGN and plasma membrane were heavily labeled with gold particles. The cellular localization of the influenza virus transgenic protein is in agreement with other reports [60,61]. The lack of gold particles on IMVs and immature viruses suggests that the IMV membrane is not derived from cellular membranes associated with the exocytic pathway.

## Conclusion

We have shown that recombinant viruses obtained from co-infection of cells with MVA vectored influenza vaccine and a naturally circulating cowpox virus displayed parental, but also potentially important non-parental characteristics, which were not predictable from the outset. A major observation is that the transgene negative viruses have enhanced multiplication compared to the transgene positive progenitor virus strain, and that the host cell type may affect the stability of the transgene or its phenotype

## Methods

### Cells, viruses, and antibodies

All cell lines were purchased from the American Type Culture Collection (ATCC). Cells (Table 1) except A549 were cultured under conditions suggested by ATCC. A549 cells were propagated in Hams F12 medium supplemented with 20% foetal bovine serum (FBS) (Invitrogen AS, Karlruhe, Germany). The CPXV-NOH1 was originally isolated from a woman with necrotic ulcer [62]. MVA-HANP was provided by Dr. Bernard Moss, National Institute of Health, USA. The genome of MVA-HANP contains influenza virus (A/PR/8/34) *HA *and *NP *inserts [63]. CPXV-NOHI and MVA-HANP were parental viruses used for co-infection of BHK-21 cells. The isolation and restriction enzyme mapping of parental and progeny hybrid viruses was reported elsewhere [6]. The progeny hybrid viruses are CPXV/MVA-Rec1 (Rec 1), CPXV/MVA-Rec2 (Rec 2), CPXV/MVA-Rec3 (Rec 3), CPXV/MVA-Rec3a (Rec 3a) and CPXV/MVA-Rec3b (Rec 3b). Rec 3a and Rec 3b are transgene negative derivatives of Rec 3 [6]. The stock of parental and progeny viruses except MVA-HANP were prepared from infected Vero cells. MVA-HANP stock was prepared from infected BHK-21 cells. Anti influenza virus HA monoclonal antibody, H28E23 was a gift from Dr. Bernard Moss.

### Immunostaining

Plaques of viruses carrying the influenza virus *HANP *transgenes were visualized by immunostaining as described previously [6]. Briefly, virus infected cells were fixed in 1:1 solution of methanol: acetone for 2 minutes. Anti influenza virus monoclonal antibody H28E23 was used as primary antibody in 1: 500 dilution. The secondary antibody, rabbit anti mouse IgG conjugated with peroxidase (Dako, Glostrop, Denmark) was used in 1:200 dilution. Both antibodies were diluted in phosphate buffered saline (PBS) supplemented with 3% FBS. DAB Peroxidase (Sigma Fast™ 3, 3^1^diaminobenzidine tablet sets) was used as substrate (Sigma Aldrich Chemie Gmbh, Steinhein Germany). This staining method was used to determine the titre of the transgene positive virus strains under low and high m.o.i.

### Plaque Assay

The titres of transgene negative viruses under low and high m.o.i were determined by plaque assay as described previously [53]. Virus was adsorbed to cell monolayer for one hour at 37°C. The inoculum was removed and infected cells were incubated in fresh medium supplemented with 2.5% FBS at 37°C in a 5% CO_2 _atmosphere. After 36 hours, the medium was removed, and the infected cells were stained with crystal violet (0.1% in 20% ethanol) for 30 minutes. The cells were washed and air-dried.

### Cell Line Permissivity Assay

Thirteen mammalian cell lines (Table 1) in 25 cm^2 ^tissue culture flasks were infected with parental and progeny viruses at a m.o.i of 0.05 pfu or IU per cell. Following adsorption for one hour, the inocula were removed and cells were washed twice in PBS. Fresh medium supplemented with 2.5% FBS were added and incubation was carried out for 72 hpi. Cells and medium (supernatant) were harvested. Viruses in the cells were released by three cycles of freeze-thawing and brief sonication. Virus multiplication and CPE were quantified as reported previously [25].

### Kinetics of virus multiplication

Vero and IEC-cell monolayers in 25 cm^2 ^TC flasks were infected with respective virus strains at low (0.01 pfu/cell) and high (5.0 pfu/cell) m.o.i respectively. After adsorption for one hour at 37°C, the inocula were removed. Infected cells were washed twice in PBS and incubated in appropriate medium supplemented with 2.5% FBS at 37°C in a 5% CO_2 _atmosphere. At multiple times post infection, cells and supernatant were harvested. Virus titre in the cell lysate and supernatant were determined by plaque assay and immunostaining.

### Transgene Stability Assay

The stability of the influenza virus *HA *insert present in the genome of the transgene positive viruses was monitored indirectly by immunostaining. Plaque purified transgene positive virus strains were passaged many times in Vero and IEC-6 cells at a m.o.i of 0.01 pfu per cell. After each passage, the infectious titre and the number of HA positive and HA negative plaques were quantified by immunostaining. Back titration of MVA-HANP was done in IEC-6 monolayers while that of other transgene positive virus strains were carried out in Vero cells.

### Transmission Electron Microscopy

Cell monolayers in six well tissue culture plates (NUNC Sweden) were infected with parental and progeny viruses at a m.o.i of 5 pfu per cell. Adsorption was for one hour at 4°C. The cells were washed thrice in PBS and incubated in fresh medium containing 2.5% FBS at 37°C in 5% CO_2 _atmosphere. At 6, 12, 18, and 24 hpi, the medium was removed. Infected cells were rinsed with fresh medium, and fixed in MacDowells solution, pH 7.4 containing 1% glutaraldehyde and 4% formaldehyde in PBS. Fixed cells were processed for electron microscopy as reported elsewhere [64]. Mature and immature forms of parental and progeny viruses were counted in 50 cell sections that were infected. Absolute and relative amount of viral forms were calculated. Collection of images from thin sections was done using JEOL 1010 electron microscope operating at an accelerating voltage of 100 KV.

### Negative Staining

Parental and progeny virions were semi-purified from infected cells by pelleting through a sucrose cushion [65]. Purified virions were negatively stained with 1% phosphotungstic acid, pH 6.2. Stained virions were adsorbed to formvar coated grids. Virions were visualized in a JEOL JEM – 1010 electron microscope. The shape and dimensions of 50 virions were determined for each of the virus strains under study.

### Cryo-immunogold Electron Microscopy

Monolayers of Vero, IEC-6, and A549 cells were infected with parental and progeny virus strains at a m.o.i of 5 pfu or IU per cell. Virus adsorption was for one hour at 4°C. Cells were washed in PBS and incubated in a fresh medium supplemented with 2.5% FBS under standard conditions. At 12 and 24 hpi, the culture medium was removed and infected cells were rinsed with PBS, fixed overnight in 4% formaldehyde – 0.1% glutaraldehyde in Hepes buffer, pH 7.4. Fixed cells were pelleted, resuspended in 10% gelatin and infiltrated in 2.3 M sucrose. Infiltrated cells were processed for pre-embedding immunogold labeling [66]. Anti-influenza virus HA monoclonal antibody H28E23 diluted 1: 400 in 1% cold water fish gelatin, CWFSG (Sigma G-7765) was used as the primary antibody. The influenza virus HA monoclonal antibody was detected with rabbit anti mouse IgG (diluted 1: 400 in CWFSG) conjugated to 10 nm gold particles. The localization of the transgenic protein in the cellular and viral structures was documented with JEOL 1010 electron microscope.

## Competing interests

The authors declare that they have no competing interests.

## Authors' contributions

MIO designed the study, carried out all experiments, and wrote the draft of the manuscript. ØN contributed to the design of the study, interpreted data and revised the manuscript. UM interpreted the data and revised the manuscript. MT interpreted data and revised the manuscript. TT conceived the study, contributed to its design, interpreted data and revised the manuscript. All authors' have read and approved the final manuscript.
